# Concerning the Inoculability of Typhoid Fever

**Published:** 1876-08

**Authors:** 


Experimental Investigations Concerning the Inoculability of Typhoid Fever—*Preliminary report by Dr. Motschuoffsky, (Odessa). From the Centralblatt fuer die medicinischen Wissenschaften, March* 11, 1876.—During the last three years, in the course of which abdominal and petechial typhus and recurrent fever have repeatedly become severe epidemics, I have taken the opportunity of carefully testing the question as to the inoculability of typhus fever. I was led to undertake these experiments by the result of the inoculation of our prosector, Dr. Munch, by the blood of a patient suffering from recurrent fever.

My experiments were made (1) on men, who of their own accord submitted to them; and (2) on animals, monkeys, rabbits, dogs, and cats. The results were as follows:

Repeated inoculations of abdominal and petechial typhus all failed, on both men and animals. Recurrent fever was quite easily inoculated into the *human* subject. The experiments on *animals* all failed.

The blood alone proved serviceable as the inoculating agent, while numerous inoculations with milk, perspiration, urine, saliva, and excrement failed.

Inoculation was successful only when the blood of the patient was taken during an attack such as those from which others were suffering. That with the blood of an apyretic patient always gave negative results.

When the blood was taken during an attack, inoculation proved successful whether the spirilli were or were not present, as for instance during the first hours of the attack.

The recurrent fever produced by artificial inoculation did not in the least degree differ from the acquired form; neither in the clinical aspects, the severity, duration, nor the number of attacks.

This fever produced by inoculation afforded fresh inoculation matter, which, however, did not confirm Davaine's theory that the inoculability is potentized by each fresh transfer.

The blood of a patient with recurrent fever produced recurrent fever exclusively, and no other form of the group of infectious diseases.

That from recurrént bilious fever caused only *recurrent* fever, but not the *bilious* variety.

The period of incubation was never less than five nor more than eight days.

The period of apyrexia corresponds closely with the period of incubation.

Inoculation with the blood taken from a person during the stage of incubation was a failure.

The quantity of blood thus introduced exerted no influence either on the duration of the period of incubation nor upon the intensity of the affection.

Blood two days old, kept in a hermetically sealed capillary tube, at a temperature of ten degrees R., gave positive results. In this case the spirilli had not lost their motility.

Inoculation with equal parts of blood and a 0.1 per cent. watery solution of quinia succeeded.

A large number of my experiments were made in the presence of my colleagues; in a future number they will be more fully detailed.—*Clinic.*

